# Investigation of High-Energy Ion-Irradiated MA957 Using Synchrotron Radiation under *In-Situ* Tension

**DOI:** 10.3390/ma9010015

**Published:** 2016-01-02

**Authors:** Kun Mo, Di Yun, Yinbin Miao, Xiang Liu, Michael Pellin, Jonathan Almer, Jun-Sang Park, James F. Stubbins, Shaofei Zhu, Abdellatif M. Yacout

**Affiliations:** 1 Nuclear Engineering Division, Argonne National Laboratory, Lemont, IL 60439, USA; diyun1979@mail.xjtu.edu.cn (D.Y.); ymiao@anl.gov (Y.M.); pellin@anl.gov (M.P.); yacout@anl.gov (A.M.Y.); 2 Department of Nuclear Engineering, Xi’an Jiaotong University, Xi’an 710049, Shaanxi, China; 3 Department of Nuclear, Plasma, and Radiological Engineering, University of Illinois at Urbana-Champaign, Urbana, IL 61801, USA; xliu128@illinois.edu (X.L.); jstubbin@illinois.edu (J.F.S.); 4 Advanced Photon Source, Argonne National Laboratory, Lemont, IL 60439, USA; almer@aps.anl.gov (J.A.); parkjs@aps.anl.gov (J.-S.P.); 5 International Institute for Carbon-Neutral Energy Research (I2CNER), Kyushu University, Fukuoka 819-0395, Japan; 6 Physics Division, Argonne National Laboratory, Lemont, IL 60439, USA; zhu@anl.gov

**Keywords:** synchrotron radiation, oxide dispersion-strengthened (ODS), ion irradiation, *in situ* tensile test

## Abstract

In this study, an MA957 oxide dispersion-strengthened (ODS) alloy was irradiated with high-energy ions in the Argonne Tandem Linac Accelerator System. Fe ions at an energy of 84 MeV bombarded MA957 tensile specimens, creating a damage region ~7.5 μm in depth; the peak damage (~40 dpa) was estimated to be at ~7 μm from the surface. Following the irradiation, *in-situ* high-energy X-ray diffraction measurements were performed at the Advanced Photon Source in order to study the dynamic deformation behavior of the specimens after ion irradiation damage. *In-situ* X-ray measurements taken during tensile testing of the ion-irradiated MA957 revealed a difference in loading behavior between the irradiated and un-irradiated regions of the specimen. At equivalent applied stresses, lower lattice strains were found in the radiation-damaged region than those in the un-irradiated region. This might be associated with a higher level of Type II stresses as a result of radiation hardening. The study has demonstrated the feasibility of combining high-energy ion radiation and high-energy synchrotron X-ray diffraction to study materials’ radiation damage in a dynamic manner.

## 1. Introduction

MA957 is a ferritic oxide dispersion-strengthened (ODS) alloy originally developed by INCO at the end of the 1970’s [1,2]. As with many ODS materials, MA957 has exceptional high-temperature strength, creep resistance, and oxidation resistance [3]. The mechanical superiority of MA957 over traditional ferritic materials stems from its unique nano-feature: a high density of nano-scale particles or nano-clusters embedded in the metallic matrix. Under irradiation, these nano-scale particles serve as traps of He and point defects, and thus significantly improve the materials’ swelling resistance [4]. MA957 is considered one of most promising structural materials for advanced reactor systems (e.g., fast reactors and fusion systems) due to its outstanding radiation resistance [5]. To better understand the properties of MA957 and further improve its mechanical performance and radiation resistance, decades have been spent performing numerous studies. The mechanical properties including tensile properties, fracture toughness, and creep resistance have been studied at various temperatures and under various test conditions [5,6,7,8]. Multiple advanced materials characterization techniques, including atom probe tomography (APT), small angle neutron scattering (SANS), and high-resolution transmission electron microscopy (HRTEM), have been employed to systematically investigate the stoichiometry, crystal structure, size, density, and stability of nano-particles/nano clusters within MA957 [9,10,11,12,13,14]. Microstructural development of MA957 after neutron radiation, an important reference for possible nuclear applications of the alloy, has been extensively studied at different temperature and doses [1,15,16,17,18,19,20].

Development of radiation resistant materials requires long-term and systematic in-reactor tests to evaluate irradiation damage as a function of temperature and dose. However, the high cost and long irradiation required for in-reactor testing hinders the progress of nuclear materials development. Recent advances in studying heavy ion irradiations have shown great potential for emulating reactor irradiation damage using ion beams [21,22]. One of the main disadvantages of using heavy ion irradiation is the limited depth of penetration with the ion beam [23]. To considerably extend the depth of ion penetration and thereby attain a larger radiation-damaged region, we employed a 84 MeV Fe ion beam, of an energy much higher than those available from lab-based accelerators. The irradiated specimen was then tensile tested with an *in-situ* synchrotron diffraction measurement. The dynamic responses of un-irradiated and irradiated regions within one single specimen were obtained. This set of experiments demonstrated the feasibility of combining high-energy ion radiation and high-energy synchrotron X-ray diffraction to study materials’ radiation damage in a dynamic manner.

## 2. Materials and Experimental Procedure

**Figure 1:**
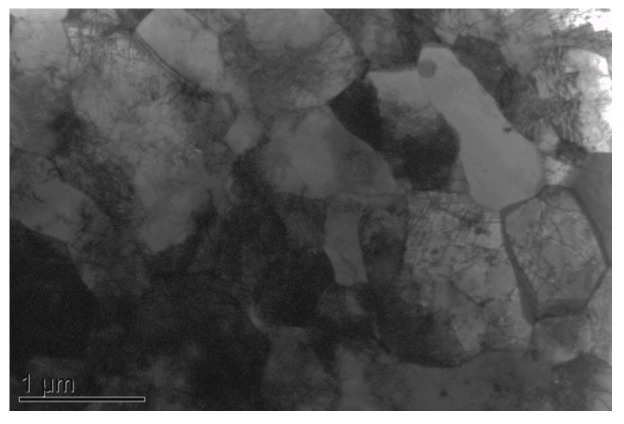
Transmission electron microscopy (TEM) image of the MA957 sample before ion-irradiations.

**Figure 2:**
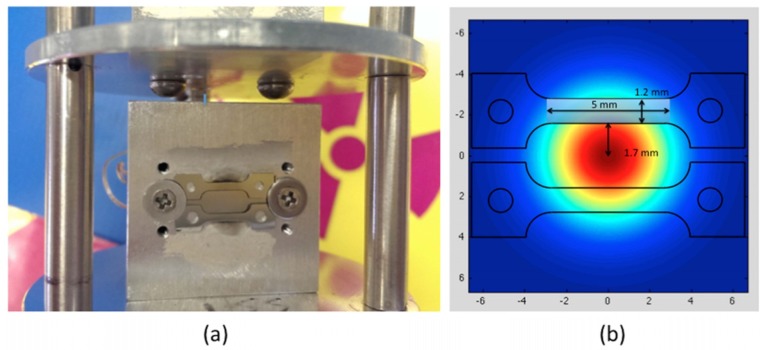
(**a**) Experimental set-up for irradiating tensile specimens at Argonne Tandem Linac Accelerator System (ATLAS); (**b**) Schematic of the Gaussian beam exposure profile of tensile specimens (axes units: mm).

The nominal composition of MA957 is 14Cr-1Ti-0.3Mo-0.25Y_2_O_3_ (wt %). The material contains ~5 nm nanoclusters [24] in grains approximately 500 nm in size. Figure 1 shows the typical microstructure of MA957. The material was machined into SS-J1 type miniature tensile specimens with a gauge section of 1.2 mm × 0.25 mm × 5 mm (width × thickness × length). The tensile specimens were polished first with 600 grit SiC abrasive paper, then by 3 μm alumina suspensions, and finished with 0.02 μm colloidal silica. The high-energy (84 MeV) Fe ion irradiation of the specimens was performed in the Argonne Tandem Linac Accelerator System (ATLAS) at Argonne National Laboratory (ANL). The Fe ion beam with a charge state of +11 was tuned to a Gaussian shape with a full width half maximum (FWHM) of 5.2 mm. The exposure area was controlled to be ~10 mm in diameter by setting up a collimator in front of the sample stage. Two specimens were loaded on the sample stage and irradiated simultaneously (Figure 2a). The large beam size allowed high-energy ion exposure of the entire gauge and part of the grip section of the miniature tensile specimens (Figure 2b). Each of the tensile specimens was placed 1.7 mm from the ion beam center in order to achieve relatively uniform radiation damage in the gauge part of the sample. The gauge region (shown as the pale blue rectangular region in Figure 2b) of each sample absorbed ~9.4% of the total beam current. The average dose rate was ~8 × 10^11^ ions/(cm^2^·s), and the achieved dose in the gauge region was ~4.4 × 10^16^ ions/cm^2^. A thermocouple was attached to the back side of each tensile specimen in order to provide continuous temperature measurement during the experiment. The stabilized sample temperature during the experiment was measured to be ~250 °C.

**Figure 3:**
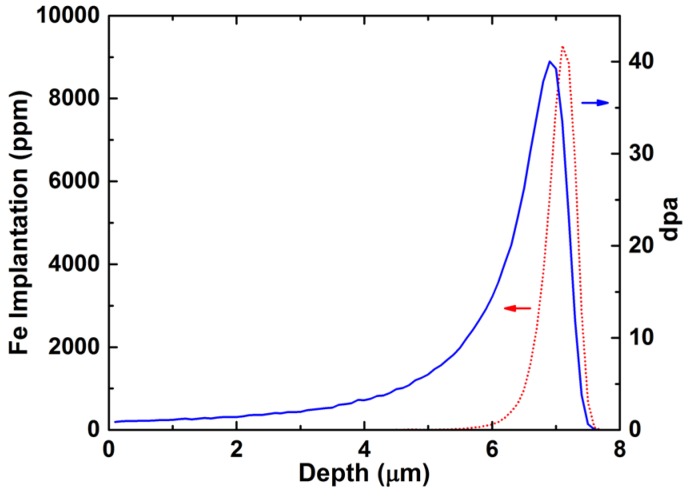
Implanted Fe ion concentration (in ppm) and radiation damage (in dpa) of the irradiated MA957 tensile sample.

Based on the average dose achieved in the gauge portion of the tensile specimens, the implanted Fe ion concentrations (in ppm) and radiation damage (in displacements per atom, dpa) were calculated by the SRIM (stopping and range of ions in matter) computer code [25,26]. A value of 40 eV displacement threshold energy and the Kinchin-Pease option were used in the SRIM calculation following the recommendations in reference [27]. The ion irradiation produced a damaged region of ~7.5 μm in depth, while the peak damage (~40 dpa) was estimated to be ~7 μm from the surface (Figure 3). The average damage levels from the surface to the depth of ~7.5 μm (over the damaged region) and 10 μm (over a single X-ray scan step) were ~7.4 and ~5.7 dpa, respectively.

**Figure 4:**
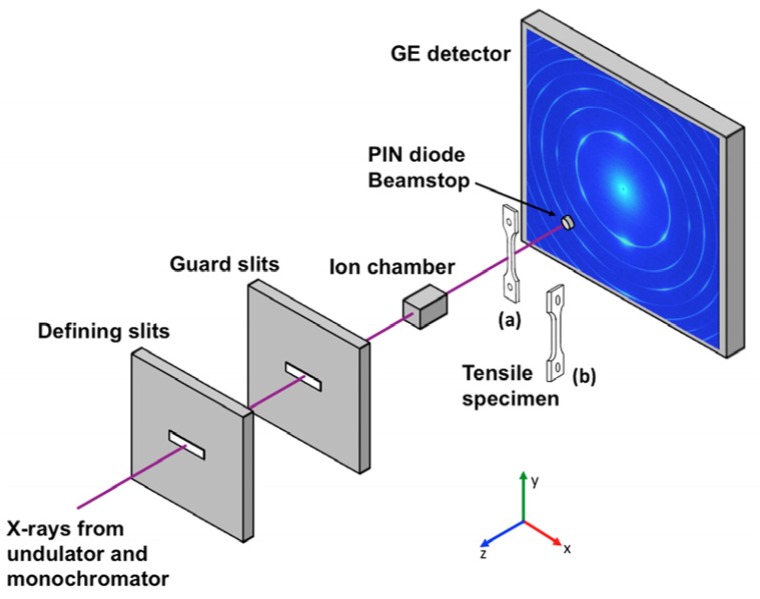
Schematic of the synchrotron experimental setup; the diffraction pattern in the schematic is from the bulk measurement of the un-irradiated MA957 tensile specimen. Sample position (a) is for bulk measurement of the un-irradiated MA957 tensile specimen; and sample position (b) is for X-ray diffraction scan of the ion-irradiated MA957 tensile specimen.

**Figure 5:**
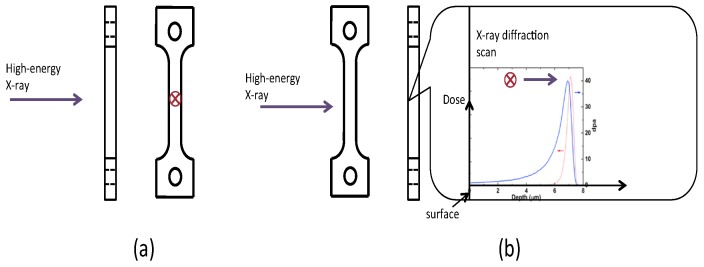
Schematic of the X-ray diffraction measurement of: (**a**) un-irradiated MA957, *i.e.*, the bulk measurement; and (**b**) ion-irradiated MA957 by in-depth cross-section scanning. The red circles with cross show the X-ray inlet direction.

The *in-situ* high-energy X-ray diffraction experiment was conducted at the 1-ID beamline of the Advanced Photon Source (APS), ANL. One of the irradiated specimens and one un-irradiated MA957 tensile specimen (as a control sample) were deformed in uniaxial tension using an MTS closed-loop servo-hydraulic test frame (model 858) at room temperature. To perform the X-ray diffraction measurements during tensile testing, the specimen was installed on the grips without any applied load from the MTS test frame. After specimen placement, a 0.1 N load (stress of ~88 MPa) was applied to fix the specimen in position. During tensile testing, the gauge part of the specimen was exposed to the high-energy monochromatic X-ray beam (86 keV), and the diffraction patterns were collected by an amorphous Si detector from General Electric (GE) with a pixel size of 200 μm. Two different types of *in-situ* measurements were performed on the two tensile specimens: (1) a continuous (non-stop) tensile test with X-ray diffraction to measure the bulk response of the un-irradiated MA957 and (2) an intermittent tensile test with X-ray diffraction scanning across the cross-section in order to capture the response in the radiation-damaged region near the sample surface of the ion-irradiated MA957. Figure 4 shows the experimental setup and the sample orientation during the *in-situ* tensile tests. A schematic of the X-ray diffraction measurement of the un-irradiated and ion-irradiated samples is shown in Figure 5. The bulk measurement of the un-irradiated specimen was done using a typical continuous *in-situ* tensile test with X-ray diffraction characterization. The strain rate was approximately 2 × 10^−4^ s^−1^ based on the displacement rate of the load frame crosshead and the length of the gauge section. The size of X-ray beam was 100 × 100 μm^2^. The distance between the sample and the detector was ~1.4 m. Similar experimental setup and procedures can be found in references [28,29,30,31,32,33]. A unique X-ray diffraction scanning routine was designed specifically to characterize the ion-irradiated specimen. After loading the specimen on the MTS machine, the surface of irradiated specimen was oriented to be parallel to X-ray beam, *i.e.*, 90° off from the orientation of the un-irradiated specimen during its continuous tensile test. To accurately align the sample, its orientation was adjusted by using the rotation stage under the MTS machine at each stress-strain state during tensile testing. This orientation adjustment relied on the measurement of attenuated X-rays when scanning the cross-section of the sample. The beam size was reduced to 10 × 10 μm^2^ using defining slits. The beam energy was set to ~65 keV to attain a better resolution, while the distance between the sample and the detector was maintained to be ~1.4 m. During the early stage of the tensile test (before sample yielding), load control mode was used to strain the sample to a preset load, then the sample was held at that load to allow X-ray diffraction scanning. The X-ray diffraction scanned from the sample surface (Figure 5b) to ~75 μm depth with a step size of 10 μm. The first step measurement was used to represent the irradiated region. Once sample yielding was observed, the load control mode was changed to strain control mode to avoid overloading the tensile specimen. This intermittent tensile test has been shown to yield similar material performance to the continuous tensile test at low temperatures, and has been applied to study materials’ behavior with *in-situ* wide-angle X-ray and neutron scattering [32,34,35,36,37,38,39,40].

## 3. Results

**Figure 6:**
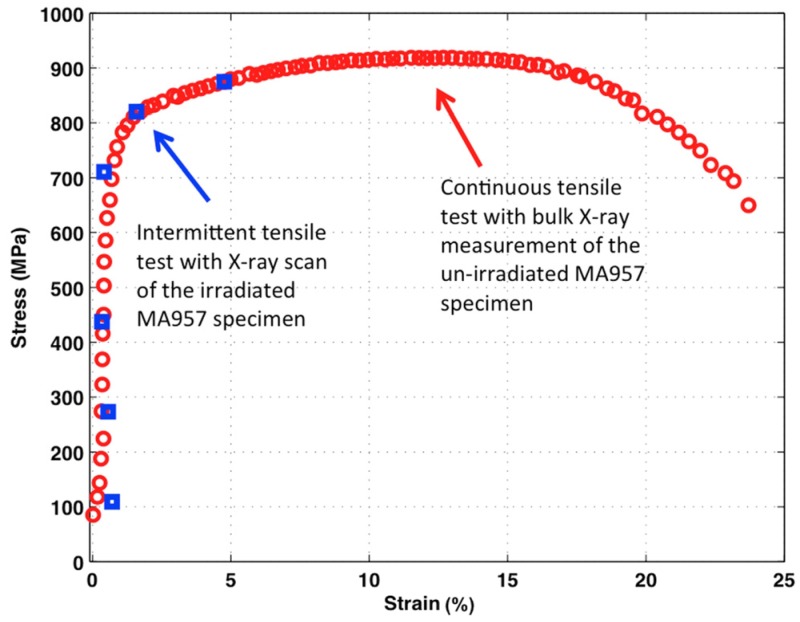
Engineering stress-strain diagram of MA957: the blue squares show stress and strain values during the intermittent tensile test for the irradiated MA957 specimen; the red circles show stress and strain values during the continuous tensile test for the un-irradiated MA957 specimen.

Figure 6 shows the stress-strain diagram of the MA957 alloy. The engineering stress, σ_e_, was calculated by σ_e_ = *F*/*A*, where *F* is the load on the sample and *A* is the area of the cross-section. *F* was measured by the MTS loading frame, while *A* was measured before the tensile test. The entire stress-strain curve was only achieved by tensile testing the un-irradiated bulk MA957 sample. The 0.2% yield strength (YS) was measured to be ~810 MPa. The ultimate tensile strength (UTS) was measured to be ~918 MPa, developed at a strain of ~13.4%. Sample necking began after the sample reached the UTS and continued until sample failure. The total elongation of the 9Cr ODS sample was ~24.1%. For the tensile test of the irradiated specimen, only six individual measurements along the stress-strain curve were conducted because the sample failed when loading to the seventh position in plastic deformation. However, all the measured points fell along the stress-strain curve developed by the continuous test, and this directly confirmed the material’s similar mechanical response for intermittent and continuous tensile tests.

**Figure 7:**
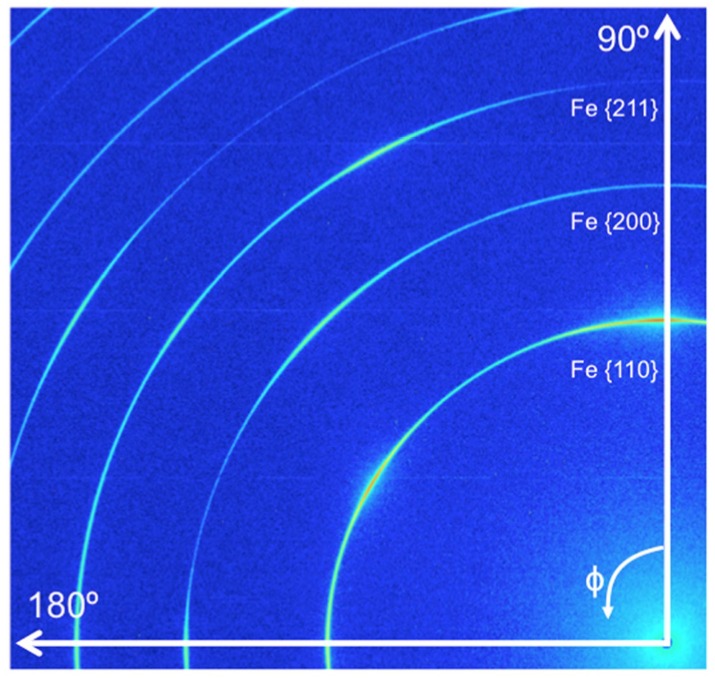
Diffraction pattern of un-irradiated MA957; the tensile direction is at 90°.

**Figure 8:**
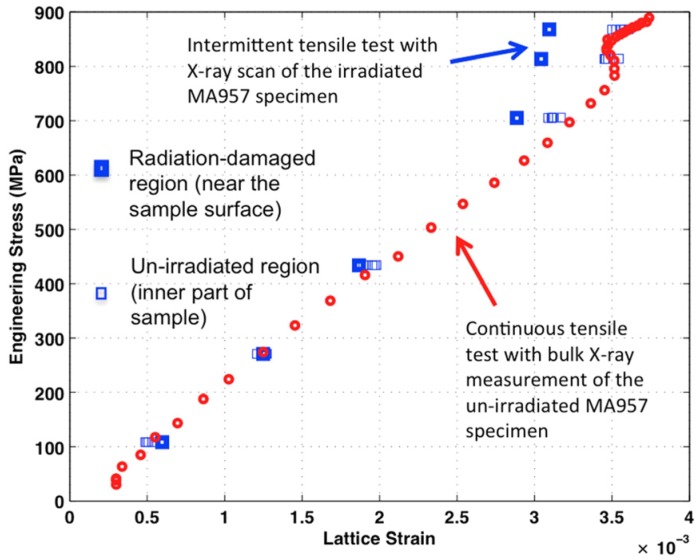
{110} lattice strain evolutions for both un-irradiated and irradiated MA957 samples: the blue squares show lattice strain values during the intermittent tensile test for the irradiated MA957 specimen; the red circles show lattice strain values during the continuous tensile test for the un-irradiated MA957 specimen. The uncertainty in lattice strains is about ±4 × 10^−4^.

Figure 7 shows the diffraction pattern of the un-irradiated MA957. A strong {110} texture was observed. The lattice constant of MA957 was measured to be 2.873 Å. Since a small beam size was used, a limited X-ray diffraction volume was obtained when measuring the ion-irradiated specimen. Moreover, due to the developed texture in the MA957 sample, only the {110} reflection of the α-Fe matrix provided sufficient statistics in the tensile direction (*i.e.*, 90° in Figure 7) when measuring the irradiated sample. To study and compare the dynamic response of both materials during tensile testing, the strain in the {110} family of planes in the α-Fe matrix was calculated based on the equation [32,41,42,43,44,45]:

(1) $$\varepsilon_{\text{lattice}}=\frac{d_{\sigma }-d_{0}}{d_{0}}$$

where *d*_σ_ is the d-spacing of a {110} reflection measured at a specific stress σ and at the 90° (*i.e.*, tensile direction) of the {110} Debye ring. The *d*_0_ is the reference d-spacing measured before loading the specimen. Figure 8 shows the results of the lattice strain evolution for both the un-irradiated and irradiated MA957 samples. As the testing was done continuously, the lattice strain of un-irradiated MA957 evolves smoothly. With applied load, the lattice strain linearly increases in the elastic regime until yielding (red circles in Figure 8). Similar to many materials with multiple phases [18,19,20,26], in the transition process from elastic to plastic deformation, the lattice strain of the metallic matrix decreases during early yielding but increases afterwards until necking. Compared to the homogeneous microstructure within the un-irradiated sample, the ion-irradiated MA957 has two regions that need to be separately characterized. By using X-ray diffraction scanning with small beam size, both the radiation-damaged region near the sample surface and the un-irradiated region at the inner part of the sample were measured; their lattice strains were calculated and are shown in Figure 8. The lattice strain development in the un-irradiated regions is consistent with the bulk measurement of the un-irradiated specimens, although the deviation from the elastic linearity was not caught due to the small number of measurements during sample yielding. During the early stage of elastic deformation, the loading behavior of the radiation-damaged and the un-irradiated regions in the irradiated sample are also consistent with the bulk measurement of the un-irradiated specimens. The divergence of the lattice strain development in the radiation-damaged region starts at a stress of 700 MPa, about 100 MPa below the yield strength. Upon plastic deformation, the difference between the lattice strain developed in the irradiated and un-irradiated regions increases significantly; its value jumped from ~0 at 430 MPa to 4 × 10^−4^ at 815 MPa. This indicates that the internal stress of the metallic matrix in the radiation-damaged region is much smaller than that in the un-irradiated region of the same specimen.

## 4. Discussion

The surface condition is critical for ion-irradiation study, especially when using a lab-based accelerator wherein the induced irradiation damage is concentrated in the near-surface region. The high-energy ions used in the present study produced a ~7.5 μm deep damage region. Thus there is much less sensitivity to the surface condition than when irradiating with lower energy ions. However, electropolishing should be applied to the specimens in further irradiation experiments to provide a smooth and hardening-free sample surface.

As shown in Figure 6, bulk stress-strain responses of the irradiated and un-irradiated regions are consistent, indicating that the impact of the high-energy ion irradiation on the overall tensile sample is negligible. Even though the Fe ion energy is much higher than regular lab-based accelerators, the radiation-damaged region is limited to <10 μm from the surface. Similar lattice strain developments in the un-irradiated sample and the un-irradiated region of the irradiated sample also suggest that the high-energy radiation damage is localized in the near-surface region. This provides us an opportunity to investigate the dynamic responses of un-irradiated and irradiated materials by applying a high-energy X-ray scan to both un-irradiated and irradiated regions within a single specimen. Note that the un-irradiated region in the irradiated sample experienced similar temperature exposure during the high-energy ion irradiation; the temperature gradient along the range of X-ray diffraction scan (surface to the 75 μm depth) was insignificant based on the heat transfer calculation. Thus, the comparison between these two regions within a sample is even better than the comparison to the un-irradiated/control sample.

The radiation effect on the tensile specimen was not evident during the early stage of elastic deformation, indicating that the elastic constant of the MA957 was not changed after high-energy ion irradiation. The difference in lattice strain between the radiation-damaged region and the un-irradiated region became significant when the specimen was loaded near the YS, where the lattice strains began to deviate from the elastic linearity. This phenomenon of deviation from elastic linearity during yielding (Figure 8) is often attributed to the development of Type II stresses [46]. Type II stresses universally exist in polycrystalline metals, and are more significant in multi-phase materials because of the larger discrepancy between phases [47,48]. Compared to the un-irradiated region, the ion-irradiated MA957 region developed higher levels of Type II stresses that resulted in a more significant deviation from the elastic linearity. The radiation induced hardening within the damaged region, caused by a high density of radiation induced defects (*i.e.*, point defects and dislocation loops), may have contributed to the increase in type II stresses when external load was applied. Another possible reason for the radiation induced hardening is the instability of nano-scale particles within the material. For example, the size of nano-scale particles can be significantly decreased after ion irradiation (the radiation damage level is less than 60 dpa) [49].

A major difficulty in this synchrotron experiment is setting the sample alignment in order to enable the high-energy X-ray to accurately penetrate the small radiation-damaged region. This alignment must be conducted at every stress-strain state during the intermittent tensile test; optimizing the sample orientation relative to the X-ray took 30–60 min. During the alignment, the tensile specimen was held by controlling load or displacement, and X-ray absorption measurements were performed periodically at different rotation-angles to search for the minimum absorption near the sample surface. This procedure is difficult to apply to the high-temperature tensile tests because the sample may creep during the alignment. To avoid a time-intensive alignment, a smaller specimen, for example, a wire sample with a diameter of 10 μm or less, would be ideal for the high-energy X-ray measurement. Another option is a thin film specimen with a thickness of 30 μm or less. In this case, the alignment time would be greatly reduced.

As shown in Figure 3, the radiation damage region is not uniform within the volume of measurement (*i.e.*, to a 10 μm depth from the sample surface). The lattice strains of the radiation-damaged region are the result of mixed and complicated responses to both injected interstitial and radiation damage that has huge variation over the region being analyzed. To interpret damage-level (or dpa level) dependent information amidst this complexity is extremely difficult. To better develop the capability to characterize radiation damage in materials by combining high-energy ion radiation and high-energy X-ray diffraction, a micron/submicron sized X-ray beam will be needed to probe into a specific region of interest in an irradiated sample. For example, an X-ray beam can be focused to <2 μm vertically at the beamline sector 1 at APS [50], and beamline sector 34 at APS can provide an X-ray beam with a beam size <500 nm [51]. These focused X-ray beams without much loss in flux will significantly benefit the future studies of materials’ ion-irradiation damage.

## 5. Conclusions

In this paper we have demonstrated the feasibility of combining high-energy ion radiation and high-energy synchrotron X-ray diffraction to study materials’ radiation damage in a dynamic manner. The high-energy ion radiation produced a relatively large radiation-damaged region that demonstrates the possibility for study of the mechanical dynamic response of the region with applied stresses. Through *in-situ* X-ray measurement during the tensile test of ion-irradiated MA957, different loading behaviors of the irradiated and un-irradiated regions were observed within the specimen. With the same amount of applied stresses, lower lattice strains were found in the radiation-damaged region compared with those in the un-irradiated region. This difference might be associated with a higher level of Type II stresses as a result of the radiation hardening in the MA957 sample.
